# Transcriptomic and epigenomic landscapes of muscle growth during the postnatal period of broilers

**DOI:** 10.1186/s40104-024-01049-w

**Published:** 2024-07-04

**Authors:** Shuang Gu, Qiang Huang, Yuchen Jie, Congjiao Sun, Chaoliang Wen, Ning Yang

**Affiliations:** 1 State Key Laboratory of Animal Biotech Breeding and Frontier Science Center for Molecular Design Breeding, China Agricultural University, Beijing, 100193 China; 2 National Engineering Laboratory for Animal Breeding and Key Laboratory of Animal Genetics, Breeding and Reproduction, Ministry of Agriculture and Rural Affairs, Department of Animal Genetics and Breeding, College of Animal Science and Technology China Agricultural University, Beijing, 100193 China; 3 Sanya Institute of China Agricultural University, Hainan, 572025 China

**Keywords:** ATAC-seq, Broiler, Pectoral muscle development, Rapid growth, RNA-seq

## Abstract

**Background:**

Broilers stand out as one of the fastest-growing livestock globally, making a substantial contribution to animal meat production. However, the molecular and epigenetic mechanisms underlying the rapid growth and development of broiler chickens are still unclear. This study aims to explore muscle development patterns and regulatory networks during the postnatal rapid growth phase of fast-growing broilers. We measured the growth performance of Cornish (CC) and White Plymouth Rock (RR) over a 42-d period. Pectoral muscle samples from both CC and RR were randomly collected at day 21 after hatching (D21) and D42 for RNA-seq and ATAC-seq library construction.

**Results:**

The consistent increase in body weight and pectoral muscle weight across both breeds was observed as they matured, with CC outpacing RR in terms of weight at each stage of development. Differential expression analysis identified 398 and 1,129 genes in the two dimensions of breeds and ages, respectively. A total of 75,149 ATAC-seq peaks were annotated in promoter, exon, intron and intergenic regions, with a higher number of peaks in the promoter and intronic regions. The age-biased genes and breed-biased genes of RNA-seq were combined with the ATAC-seq data for subsequent analysis. The results spotlighted the upregulation of *ACTC1* and *FDPS* at D21, which were primarily associated with muscle structure development by gene cluster enrichment. Additionally, a noteworthy upregulation of *MUSTN1*, *FOS* and *TGFB3* was spotted in broiler chickens at D42, which were involved in cell differentiation and muscle regeneration after injury, suggesting a regulatory role of muscle growth and repair.

**Conclusions:**

This work provided a regulatory network of postnatal broiler chickens and revealed *ACTC1* and *MUSTN1* as the key responsible for muscle development and regeneration. Our findings highlight that rapid growth in broiler chickens triggers ongoing muscle damage and subsequent regeneration. These findings provide a foundation for future research to investigate the functional aspects of muscle development.

**Supplementary Information:**

The online version contains supplementary material available at 10.1186/s40104-024-01049-w.

## Introduction

Chicken, a pivotal source of animal high-quality protein in human diets, stands as one of the most widely consumed food products globally [1]. Over the past few decades, a substantial surge in body weight and pectoral muscle weight has been witnessed, mainly attributable to intensive genetic selection [2, 3]. Skeletal muscle, accounting for about 40% of body mass of animals, is comprised of elongated, multinucleated muscle fibers [4, 5]. Multinucleated muscle fibers are formed from the fusion of myoblasts during embryonic myogenesis [6]. The number of myofibers in animals typically remains unchanged after birth, with muscle growth primarily depending on myofiber hypertrophy [7]. The growth and development of skeletal muscle is a complex biological process that is regulated by diverse genes, transcription factors, and epigenetic factors [8]. Revealing the molecular mechanisms of animal muscle development has been a focus of research in the field of genetic and breeding research.

With the advancements in high-throughput sequencing and multi-omics technology, unraveling the intrinsic biological mechanisms of muscle development has become more convenient and cost-effective [9, 10]. An increasing number of genes or quantitative trait loci that regulate the growth traits of chickens have been characterized. A recent analysis from genome-wide association studies (GWAS) identified *ADGRG6* as a major candidate gene responsible for carcass composition traits [11]. The genes *NSUN3* and *AGK* were related to muscle development in broiler chickens. Despite the decreasing sequencing costs, GWAS remain costly, necessitating large sample sizes to gain sufficient SNP data [12, 13]. Challenges like linkage disequilibrium and population stratification complicate the accurate identification of causal variants in GWAS [14]. Further research at the transcriptome level could provide more comprehensive mechanistic insights [15, 16]. Comparison studies between modern broiler chickens and local breeds, conducted through RNA sequencing have revealed the upregulated genes such as *IGF1*, *IGF1R*, *WFIKKN2*, positively regulating chicken growth [17]. In another study using fast- and slow-growth meat chickens, DNA methylation analysis identified methylation sites in growth factors like *IGF1R* and *FGF18*, which are related to skeletal muscle development [18]. In 2013, the assay for transposase accessible chromatin with high-throughput sequencing (ATAC-seq) was developed for evaluating genome-wide chromatin accessibility and identifying transcription factor binding sites [19]. Integrating ATAC-seq with high-throughput paired-end RNA sequencing (RNA-seq) data can offer more precise information on the impact of possible regulatory differences in differentially enriched open chromatin regions [20]. This combined approach provides a valuable avenue for exploring the regulatory landscape influencing important traits in broiler chickens.

It is noteworthy that there are distinctions in the skeletal muscle development between laying hens and broilers. Even under optimal feeding conditions, the weight of 6-week-old broiler chickens is more than five times that of laying hens [21, 22]. This significant difference leads to considerable physiological and genetic regulatory changes in broiler chickens. Previous research has indicated that the first 3 weeks after hatching of broilers are crucial for metabolic adjustment, during which the liver’s metabolism shifts to process a carbohydrate-rich diet to support the rapid overall growth of modern broilers [23]. Despite certain strides in understanding broilers, there is still limited exploration of genetic regulation changes during the rapid growth phase of broilers. However, uncovering these changes has the potential to provide valuable insights for further enhancing broiler production efficiency.

To gain a deeper insight into this progression, we explored the transcriptional and epigenetic changes that take place during postnatal muscle development by RNA-seq and ATAC-seq. This study presents a finely detailed developmental profile of broiler pectoral muscle at the levels of gene expression and chromatin accessibility, identifying numerous candidate genes and unveiling functional relationships between them. Through integrated analysis, it has come to light that broiler chickens exhibit a persistent state of muscular injury and subsequent regenerative repair during the rapid growth phase. These findings provide crucial insights into the patterns of postnatal muscle development in broiler chickens, laying a theoretical foundation for future in-depth investigations.

## Materials and methods

### Experimental animals and phenotypic determination

The fast-growing breeds Cornish (CC) and White Plymouth Rock (RR) were used in this study. CC and RR are separately used as the paternal and maternal lines for Beijing Huadu Yukou Poultry Breeding Co. Ltd. The experimental sample numbers are showed in Additional file 1: Table S1. A total of 404 broiler chickens (192 CC and 212 RR) were weighted on the day of hatching (DOH), and 27 of them (16 CC and 11 RR) were slaughtered to accurately measure the pectoral muscle weight (PMW). To minimize potential confounding effects, the broiler chickens in the two experimental groups were randomly assigned and housed in cages. They were provided with water and food ad libitum under identical conditions. The temperature and illumination conditions were maintained according to the guidelines provided in the Huadu Yukou broiler management handbook throughout the entire growth period.

After a 12-h fasting period, the broiler chickens were slaughtered on various days: day 7 after hatching (D7), D14, D21, D28, D35 and D42. The data for body weight (BW) and PMW were obtained from different numbers of broiler chickens in the CC group and the RR group. Specifically, 12, 15, 15, 15, 15, and 91 broiler chickens were used in the CC group at D7–D42, while 12, 15, 15, 15, 15, and 86 broiler chickens were used in the RR group. For myofiber morphology determination, pectoral muscle samples were collected from the right side of a total of 224 individuals on D7 (12 CC and 12 RR), D14 (15 CC and 15 RR), D21 (15 CC and 15 RR), D28 (15 CC and 15 RR), D35 (15 CC and 15 RR) and D42 (40 CC and 40 RR). Additionally, the left side of the pectoral muscle samples from six individuals, randomly selected from each breed, were immediately collected post-slaughter at D21 and D42. These samples were frozen in liquid nitrogen for RNA-seq, and three biological replicate samples were used for ATAC-seq.

### Paraffin section preparation and myofiber detection

Muscle samples underwent fixation with 4% paraformaldehyde for 48 h, followed by dehydration using graded ethanol solutions and paraffin embedded. Subsequently, the wax blocks were transversely sectioned (with a thickness of 10 µm), after which the sections were subjected to hematoxylin–eosin (H&E) staining. To capture images, the stained sections were scanned using the high-resolution scanner (Pannoramic MID, 3DHISTECH, Hungary). Eight randomly selected vision fields, containing muscle fibers from the whole slide images, were chosen and imported into MyoV software [24] for the measurement of the average muscle fiber cross-sectional area (CSA) and muscle fiber density (MFD). Data analysis was conducted using R software (v.4.2.3), with results expressed as the mean ± SEM. Statistical analysis employed the one-way analysis of variance (one-way ANOVA), and differences were considered to be statistically significant at a *P* value < 0.05.

### RNA and ATAC library preparation and sequencing

Total RNAs from pectoral muscle tissues were extracted using the Eastep™ Super Total RNA Extraction Kit (LS1040, Shanghai Promega, Shanghai, China) and assessed for integrity with the Agient2100 Bioanalyzer (LabChip GX). Only RNAs with RNA integrity number greater than 6 were used for library preparations. Libraries were generated using Hieff NGS Ultima Dual-mode mRNA Library Prep Kit (13533ES96, Shanghai, China) with Hieff NGS DNA selection Beads (Superior Ampure XP alternative) (12601ES56, Shanghai, China) for PCR products purification. Subsequently, the libraries were sequenced on the Illumina NovaSeq 6000 platform.

Three biological replicates of pectoral muscle for each stage were flash-frozen in liquid nitrogen and pulverized using tissue lysates. The grinded powder was treated with cell lysis buffer and the nucleus was isolated through centrifugation for 5 min at 2,000 × *g*. The cell pellets were then resuspended in 50 µL of transposition solution and incubated at 37 °C for 30 min. Subsequently, 2×DNA clean beads were employed to purify the DNA for the PCR amplification of transposed fragments with 15 cycles. Transposition and high-throughput DNA sequencing library construction were performed using TruePrep DNA Library Prep Kit V2 for Illumina kit (Catalog No. TD501, Vazyme). The library products were enriched, quantified and finally sequenced on a Novaseq 6000 sequencer (Illumina, San Diego, California, USA) with PE150 model.

### RNA-seq data analysis

To obtain high-quality reads before moving onto subsequent analysis, the sequencing adapters and low-quality nucleotides were removed from the raw RNA-seq data using fastp (v.0.20.1) [25]. Index of the reference genome (GRCg7b) was built using HISAT2 (v.2.2.1) [26] and paired-end clean reads were aligned to the chicken reference genome (GRCg7b) using HISAT2 (v.2.2.1). The sequencing statistics of reads and bases obtained in each sample and mapping statistics of clean reads to the reference genome for each sample are summarized (Additional file 1: Table S2). The mapped reads were counted at gene level using featureCount (SUBREAD package; v.1.6.3) [27] with the chicken reference (GRCg7b) and normalized using TPM procedure to compute the TPM values for subsequent analysis steps. Principal component analysis (PCA) was performed using R package FactoMineR (v.2.9.0) [28]. Differentially expressed genes (DEGs) between different groups were identified by DESeq2 package (v.1.36.0) [29] in the R program. Genes with an adjusted *P* value < 0.05 and a log_2_ (Fold Change) > |1| were considered significant DEGs [30–32]. We referred to the DEGs that were up-regulated in CC among the breed-biased genes as CC-biased DEGs (CBGs). Similarly, the DEGs that were up-regulated in RR were referred to as RR-biased DEGs (RBGs). Among the age-biased genes, we categorized the DEGs that were up-regulated at D21 as D21-biased DEGs (D21BGs), and the DEGs that were up-regulated at D42 as D42-biased DEGs (D42BGs). Subsequently, Gene Ontology (GO) function and Kyoto Encyclopedia of Genes and Genomes (KEGG) pathway enrichment analysis were performed using the clusterProfiler package (v.4.7.1.3) in R [33].

### ATAC-seq data processing

Trimming of raw sequencing data was performed using Trimmomatic (v.0.36) [34] and then the clean reads were aligned to the chicken genome (GRCg7b) assembly using Bowtie2 (v.2.2.6) [35] with default parameters. Our sequencing exhibited showed high quality, evidenced by robust mapping rates and peak calling results (Additional file 1: Table S3). Subsequently, duplicate reads were marked and removed using Sambamba (v.0.7.1) [36]. The insert length was directly calculated from the aligned BAM file with Samtools (v.0.1.11) [37]. For peak calling, MACS2 (v.2.2.7.1) [38] was employed. IDR (v.2.0.3) was used to filter enriched regions at an IDR of less than 0.05 to generate high-confidence peaks [39]. Peak annotation and analysis were carried out using ChIPseeker (v.1.32.1) [40]. Promoter regions were defined as peaks overlapping a region that was ± 3 kb from the transcriptional start site (TSS). Differential peak analysis was performed using the diffBind package [41] with the false discovery ratio < 0.05 and log_2_ (Fold Change) > |0.5| in two comparison groups [42–44]. The Bedtools (v.2.30.0) [45] suite was employed to calculate overlap and enrichment between different intervals. The peaks of different groups were visualized in IGV tool (v.2.13.2) [46, 47].

### Integrative analysis of RNA-seq and ATAC-seq data

Since chromatin accessibility is closely related to gene regulation, the VennDiagram package (v.1.7.3) [48] in R was used to obtain the overlapping genes of DEGs derived from RNA-seq and genes associated with differential accessible regions (DARs) identified in ATAC-seq. Subsequently, the clusterProfiler [33] was utilized for GO enrichment function analysis and KEGG pathway analysis of these overlapping genes. The genes of top 10 terms were extracted for frequency statistics. We combined the genes with higher frequencies into gene sets, which were used as input for signal pathway and network analysis on the GeneMANIA [49] and STRING online platforms [50].

## Results

### Time-course growth performance analysis of broilers

**Fig. 1:**
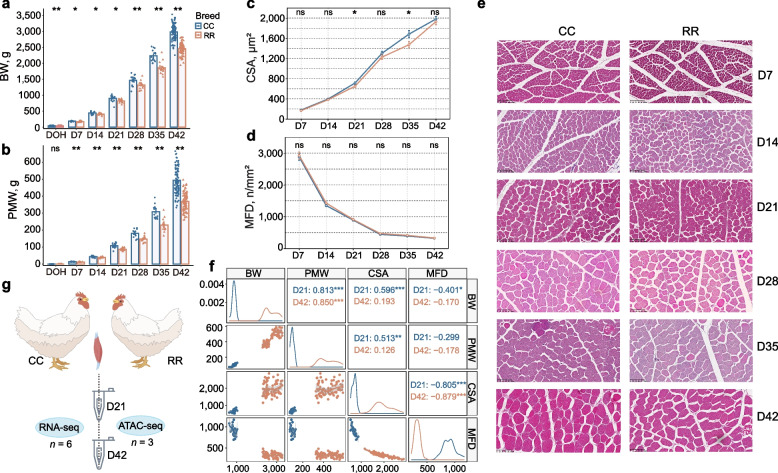
Growth performance of postnatal broiler chickens. **a** The changes in body weight (BW) of individuals with Cornish (CC) and White Plymouth Rock (RR) from the day of hatching (DOH) and day 7 after hatching (D7), D14, D21, D28, D35, D42. **b** The changes in pectoral muscle weight (PMW) of individuals with CC and RR from DOH to D42. **c** Cross-sectional area (CSA) of muscle fibers from D7 to D42. **d** Muscle fiber density (MFD) changes from D7 to D42. **e** Morphology of the myofibers stained by hematoxylin–eosin from D7 to D42. The corresponding scale is marked in the lower left corner of each image. Scale bars: 100 μm. **f** The scatter plot and Pearson’s correlation of broiler chickens at D21 and D42. **g** Pectoral muscles were collected at D21 and D42 for RNA-seq and ATAC-seq analysis. ^**^ and ^*^ indicate *P* value less than 0.01 and 0.05, respectively

We monitored the growth performance of CC and RR over a 42-d period, including the BW and PMW, and examined their morphology of muscle fiber from D7 to D42. As expected, the results showed that BW and PMW of the two groups gradually increased with advancing age while the weight of CC was significantly higher than RR at all growth stages (Fig. 1a and b, Additional file 1: Table S4). The BW and PMW of CC group were 2,986.70 ± 25.61 g and 493.76 ± 76.13 g at D42. The BW and PMW of RR were 2,460.93 ± 168.09 g and 369.03 ± 52.95 g at the same time. The histological examination revealed minor differences between CC and RR groups with CSA and MFD (*P* > 0.05) except the CSA at D21 and D35 (Fig. 1c and d) while the slice diagram revealed that the size of muscle fibers continued to increase with age. Morphology of the myofibers at all time point after hatching is shown here (Fig. 1e). The BW and PMW were positively correlated with CSA while negatively correlated with MFD of myofibers at D21 and D42 (Fig. 1f). When considering the weight results, it became evident that both BW and PMW showed a gradual increase before D21, followed by a linear increase after D21. These results suggested that D21 and D42 are crucial stages for broiler growth. To identify the genes that were differentially expressed between different groups, pectoral muscle tissue of CC and RR was collected at D21 and D42 for RNA-seq and ATAC-seq (Fig. 1g).

### Transcriptional profiling of pectoral muscle

**Fig. 2:**
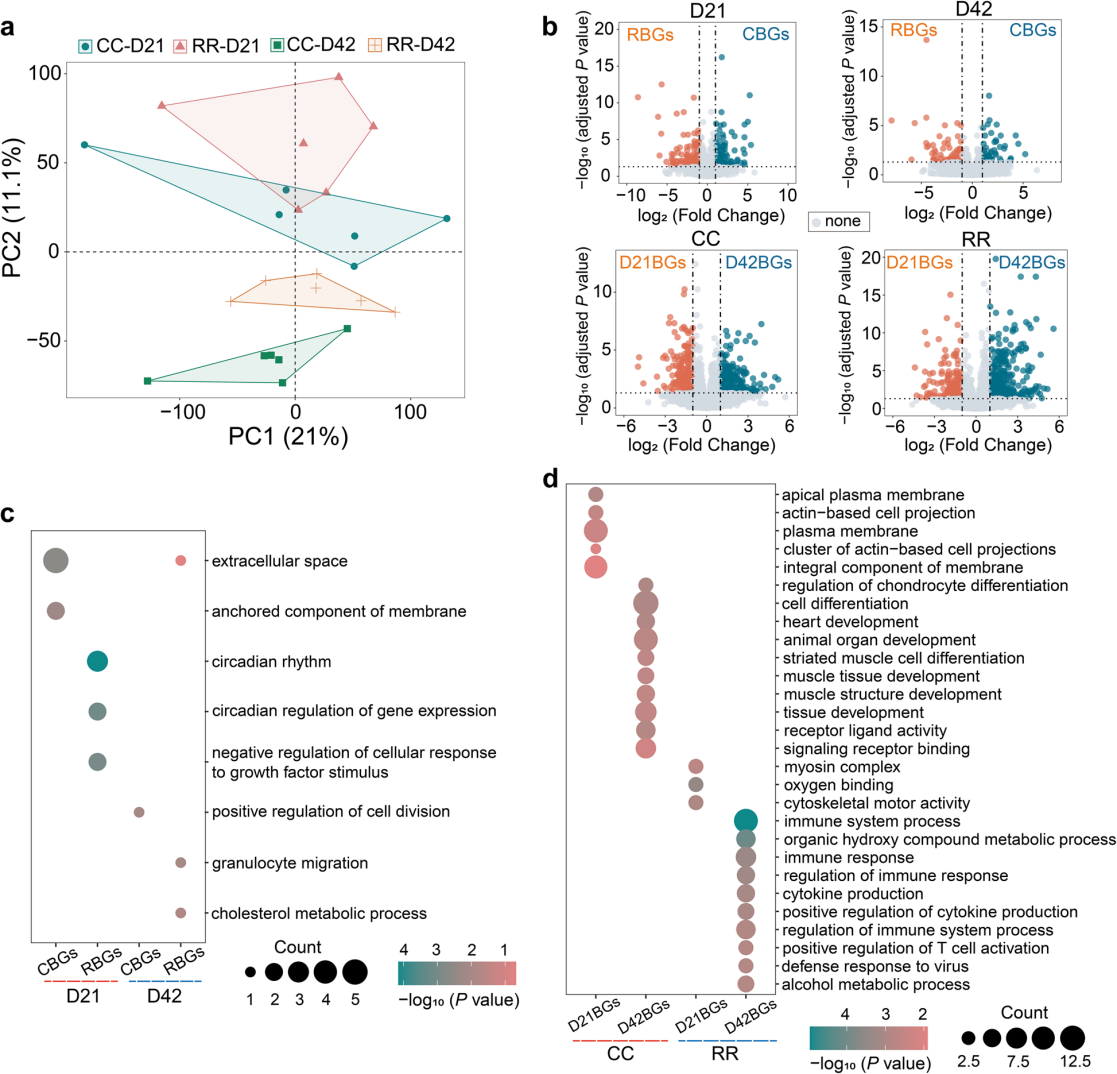
Analysis of RNA-seq data from the pectoral muscle. **a** PCA plot of RNA-seq data of CC and RR groups at D21 and D42. Each point represents a sample. **b** Volcano plot of differential expressed genes (DEGs) obtained through the comparison between pairwise groups. **c** The GO enrichment results of CC-biased DEGs (CBGs) and RR-biased DEGs (RBGs) at D21 and D42. **d** GO enrichment results of D21-biased DEGs (D21BGs) and D42-biased DEGs (D42BGs) of CC and RR

The PCA plot exhibited a separation between the two stages of D21 and D42 along PC2 (Fig. 2a). To identify the genes that were DEGs between different groups, we performed a differential expression analysis with the four groups in the two dimensions of breeds and ages. The volcano map preliminarily showed the expression of DEGs, as shown in Fig. 2b. A total of 204, 219 D21BGs and 302, 404 D42BGs of CC and RR were identified, respectively (Additional file 1: Table S4–5). Additionally, there were 137, 58 CBGs and 143, 60 RBGs at D21 and D42 (Additional file 1: Table S5–6).

In terms of differential gene functions between CC and RR groups at D21 and D42, various biological functions were enriched. At D21, CBGs were primarily enriched in terms of extracellular space and anchored component of membrane (Fig. 2c). Additionally, they were also enriched in Glycolysis/Gluconeogenesis, Fructose and mannose metabolism, Purine metabolism, and Cell adhesion molecules (Additional file 2: Fig. S1a). Among them, *ALDOB* were found to be related to both the extracellular space term and the Glycolysis/Gluconeogenesis pathway. RBGs at D21 were enriched in the terms such as circadian rhythm and negative regulation of cellular response to growth factor stimulus (Fig. 2c). The CBGs at D42 were enriched in ATP-dependent chromatin remodeling and WNT signaling pathway as shown in Fig. S1a. Furthermore, the pathway of Purine metabolism, including *NPR1*, was identified at both CBGs at D21 and D42. The RBGs at D42 were primarily enriched in cholesterol metabolic process term and amino acid metabolism pathways (Fig. 2c, Additional file 2: Fig. S1a).

A total of 137, 58 CBGs and 143, 60 RBGs at D21 and D42 were identified, respectively (Additional file 1: Table S7–8). Significant enrichment was observed in terms associated with apical plasma membrane and actin-based cell projection of D21BGs of CC (Fig. 2d). The D42BGs of CC were mainly involved in cell differentiation and muscle development, including *FOS*, *MUSTN1*, *TGFB3*, *WNT9A,* and *GREM1* (Fig. 2d). Additionally, the D21BGs of RR were enriched in terms such as myosin complex and cytoskeletal motor activity that included *MYH1D*, *MYO1A*, and *ACTC1*. The D42BGs in RR exhibited enrichment in defense response and immune system processes, as well as pathways such as cytokine-cytokine receptor interaction and cell adhesion molecules (Fig. 2d, Additional file 2: Fig. S1b). Notably, D21BGs and D42BGs of CC and RR were involved in steroid biosynthesis and cell–cell interactions (Additional file 2: Fig. S1b).

### Analysis of chromatin accessibility in pectoral muscle

**Fig. 3:**
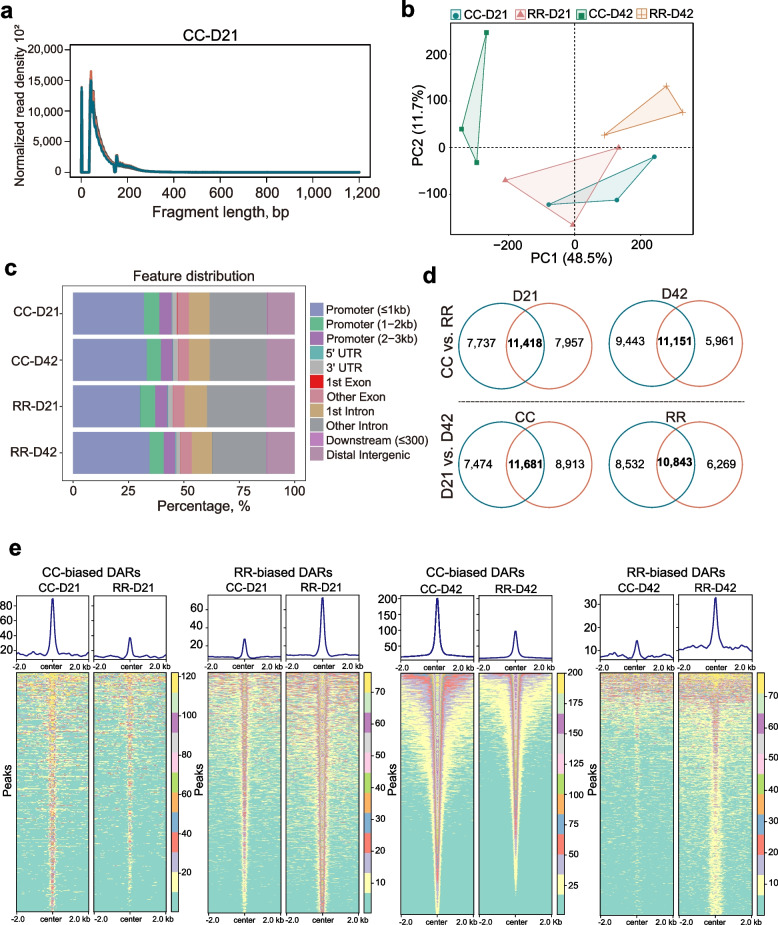
Analysis of chromatin accessibility from the pectoral muscle. **a** Fragment insert length distribution plots of ATAC-seq samples. **b** PCA plot of ATAC-seq data of CC and RR groups at D21 and D42. Each point represents a sample. **c** The feature distribution of ATAC-seq peak dataset. **d** Overlap peaks between two groups. **e** Heatmaps of differential accessible regions (DARs)

In order to better understand the mechanisms underlying gene expression alterations, we conducted a comprehensive examination of chromatin openness using ATAC-seq across the four experimental groups. The ATAC-seq data displayed approximately 200 bp fragment insert size (Fig. 3a, Additional file 2: Fig. S2a–c), and the PCA revealed a separation tendency among the four groups (Fig. 3b), indicating the reliability of the ATAC-seq data. For the 12 pectoral muscle samples, a total of 75,149 ATAC-seq peaks were identified (Additional file 2: Fig. S2d). These peaks were annotated in their promoter, exon, intron and intergenic regions, and a higher abundance observed in promoter and intronic regions (Fig. 3c). Contrasting the peaks in terms of breed and time dimensions revealed a comparable number of intersecting peaks between the two groups. Specifically, the intersection of peaks between CC and RR at D21 and D42 were 11,418 and 11,151, respectively (Fig. 3d). Furthermore, the intersection of peaks between pectoral muscle samples of D21 and D42 in the CC and RR groups were 11,681 and 10,843, respectively (Fig. 3d).

Subsequently, a pairwise comparative analysis based on the ATAC-seq data was conducted to identify DARs. A total of 3,283, 24,417, 7,916, and 15,553 significant DARs were identified in D21, D42, CC and RR development (Additional file 1: Table S9–12). The genes nearest DARs were annotated, revealing 1,486, 8,462, 3,028, and 5,615 genes at D21, D42, CC, and RR, respectively. The heatmaps revealed that these significant DARs showed very clear specific patterns in each group (Fig. 3e, Additional file 2: Fig. S2e).

### Integration analysis of ATAC-seq and RNA-seq

**Fig. 4:**
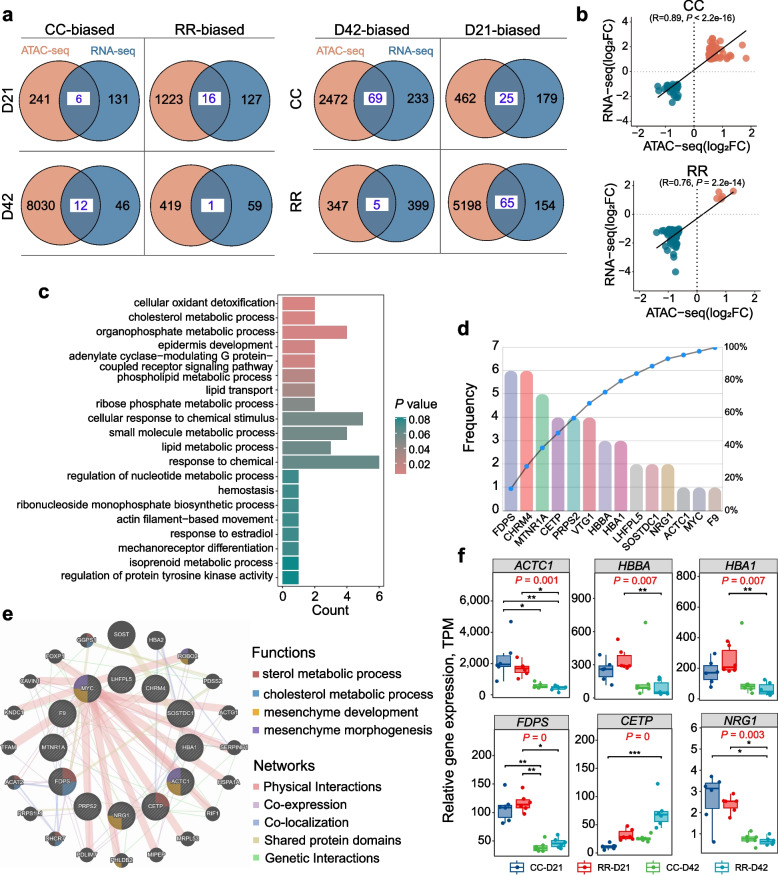
Integration of RNA-seq and ATAC-seq to identify candidate genes. **a** Venn diagrams showed the DEGs carrying DARs. **b** The relationship between DEGs and DARs. **c** The 16 RBGs at D21 and the 65 D21BGs of RR were combined for GO enrichment analysis. **d** Frequency analysis of DEGs of top 20 terms. **e** The network analysis and functional enrichment analysis of the DEGs was performed using GeneMANIA. **f** Differential expression results among the four groups. ^**^ and ^*^ indicate *P* value less than 0.01 and 0.05, respectively

To further determine the relationship between gene expression and open chromatin regions, we examined the association between gene accessibility and gene expression during pectoral muscle development by integrating RNA-seq and ATAC-seq data (Additional file 1: Table S13). As the Venn diagrams shown, we found 6 upregulated CBGs carrying DARs, including *TMEM164*, *FSTL4*, *ZNF692*, *KIF14*, *APOA5*, and *KIFC1*, and 16 RBGs carrying DARs related to muscle development, such as *MYO7B* at D21 (Fig. 4a). In addition, 12 upregulated and 1 downregulated DEGs contained DARs were identified in the CC vs. RR at D42. Among these, the RBG was identified as *IRF9*, which was associated with the inflammatory response. Focusing on the impact of age on chromatin accessibility, we identified 69 D42BGs carrying DARs of CC group, including *WNT9A*, *TGFB3* that related to muscle development. Additionally, we found 25 D21BGs containing DARs in CC groups, which included *NRG1*, a gene related to lipid metabolism, and *TMEM164*, a member of the transmembrane protein family. Moreover, 5 upregulated DEGs carrying DARs were found between D21 and D42 of RR, including *IRF7*, *DCSTAMP*, *RGCC*, *TTC7A* and *NR1D1*. In this comparison, there were 65 DEGs with DARs were downregulated at D42. As expected, a positive correlation was found between accessibility signatures and gene expression patterns, based on the calculated fold changes by assigning open chromatin regions to the nearest DEGs (Fig. 4b, Additional file 2: Fig. S2f).

### Insights into muscle development at D21 in broilers

Based on the data showed in Fig. 4a, it was determined that there were four distinct classes of DEGs exhibiting higher count level, including CC-biased genes at D42, RR-biased genes at D21, D42-biased genes of CC, and D21-biased genes of RR, within the eight identified clusters. Considering the differential expression patterns, we have merged a set of 16 RR-biased genes at D21 with 65 D21-biased genes of CC. This combination of DEGs, resulting in a total of 81 DEGs that were highly expressed in RR at D21, for subsequent analysis. Enrichment analysis indicated that these genes were mainly involved in biological processes such as cholesterol metabolic process and actin filament-based movement (Fig. 4c). For further analysis, the DEGs of top 20 terms were chosen for frequency analysis, and the 14 genes were ranked (Fig. 4d). It was observed that *FDPS* and *CHRM4* had the highest frequency, appearing 6 times, followed by *MTNR1A*, *CETP*, *PRPS2*, *VTG1*, *HBBA* and *HBA1*, indicating their crucial role in the development of RR at D21. The subsequent analysis was conducted using the GeneMANIA, which confirmed the strong association of *ACTC1*, *NRG1*, and *MYC* with mesenchyme development, and *FDPS* and *CETP* with sterol metabolic process (Fig. 4e).

The gene expression profiles in broiler chickens at both D21 and D42 are depicted in Fig. 4f. The findings indicated a significant upregulation of *ACTC1*, *HBBA*, *HBA1*, *FDPS*, and *NRG1* in broiler chickens at D21 compared to D42. It is known that the rapid muscle development occurs in broiler chickens during the D21 period. Based on these observations, it can be hypothesized that *ACTC1* and *NRG1* play crucial roles in muscle development during this period, while *FDPS*, *HBBA* and *HBA1* may exert a coordinated regulatory influence on muscular development.

### Muscle regulation dynamics and regeneration mechanisms at D42

**Fig. 5:**
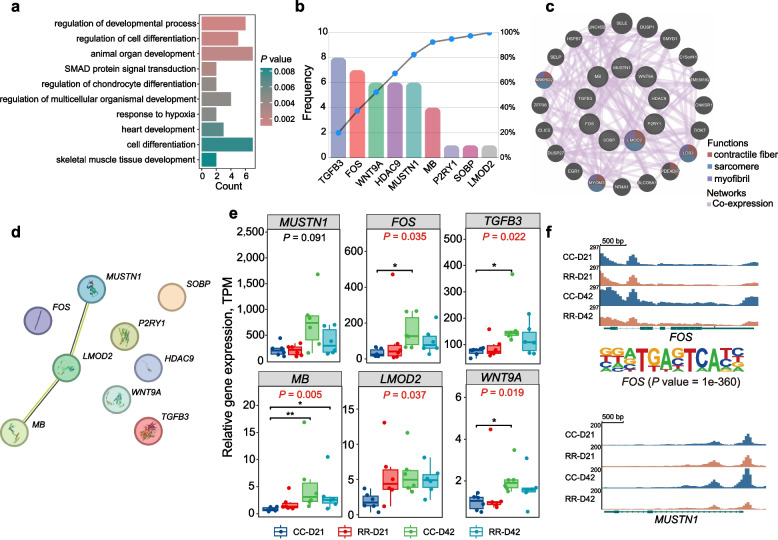
Intense muscle damage with rapid muscle growth at D42. **a** The 12 CBGs at D42 and the 69 D42BGs of CC were combined for GO enrichment analysis. **b** Frequency analysis of 9 DEGs of top 10 terms. **c** Network analysis and functional enrichment analysis using GeneMANIA on the 9 DEGs that were present in at least 2 terms among the 20 DEGs. **d** The network analysis of DEGs was performed using STRING. **e** Differential expression results among the four groups. ^**^ and ^*^ indicate *P* value less than 0.01 and 0.05, respectively. **f** Gene expression and chromatin accessibility of *FOS* and *MUSTN1* displayed similar dynamic changes in CC at D42

On the flip side, we noted that the 12 CBGs at D42 and 69 D42BGs of CC could be classified into a singular category that were highly expressed in CC at D42. This classification facilitated an in-depth exploration of the regulatory patterns governing muscle development in broiler chickens at D42, resulting in the identification of 81 DEGs. Subsequent functional enrichment analysis of these 81 DEGs revealed their significant involvement in organ developmental process, cell differentiation, and SMAD protein signal transduction (Fig. 5a). Using the method described above, we selected the top 10 terms for frequency analysis of the DEGs, and the resulting 9 genes were ranked accordingly (Fig. 5b). Notably, *TGFB3* exhibited the highest frequency, appearing 8 times, followed by *FOS*, *WNT9A*, *HDAC9*, and *MUSTN1*, underscoring their pivotal role in the development of CC at D42.

Utilizing GeneMANIA, we conducted the network analysis and functional enrichment analysis on the 9 DEGs in Fig. 5c. The results revealed that *LMOD2* was associated with myofiber contraction and cytoskeletal organization. Protein–protein interaction analysis revealed that *LMOD2* interacts with *MUSTN1* and *MB* (Fig. 5d). This finding suggested a potential functional relationship between these DEGs, which could be significant in the context of muscle development and function. Notably, we observed that *MUSTN1*, *FOS*, *TGFB3*, and *WNT9A* were highly expressed in CC at D42 among the four groups (Fig. 5e). Furthermore, the gene expression and chromatin accessibility of *FOS* and *MUSTN1*, which are related to muscle regeneration, exhibited similar dynamic changes in CC at D42 (Fig. 5f).

## Discussion

As a crucial tissue in meat-producing animals, skeletal muscle plays a vital role in initiating movements, supporting respiration, and maintaining homeostasis [51, 52]. Chickens serve as significant scientific models for studying skeletal muscle development in animals, given that the progression of chicken anatomy closely mirrors that of mammals [53, 54]. Through prolonged selective breeding practices, broiler chickens have developed an exceptionally rapid growth rate, primarily attributed to the prioritization of the pectoral muscle’s weight as a breeding goal, which rapidly increases in size after hatching [55, 56]. Modern broiler breeds are predominantly derived from the larger-sized Cornish as the sire breed and the highly productive White Plymouth Rock as the dam breed. Given this breeding background, it is imperative to investigate muscle development mechanisms in both the broiler breeds during the rapid growth phase after hatching, enabling the application of new technological approaches to cultivate broiler breeds that better meet people’s demands.

The broiler chickens were raised for marketing and consumption until D42. The primary objective of raising broiler chickens is to maximize their weight gain within a short timeframe [57]. This study demonstrated a progressive increase in body weight, aligning with common knowledge and highlighting the exceptional performance of the broiler chickens utilized in this research. Pectoral muscles represent a valuable source of high-quality protein for human consumption, and the pectoral muscle weight is a crucial economic trait in broiler chicken production [56, 58]. The growth pattern of pectoral muscle weight in this study corresponded with the observed differences between groups and body weight, with the CC group exhibiting higher values than the RR group. CC and RR have been widely used as the parental breeds for common broilers globally [59]. CC and RR are commonly used as the paternal and maternal lines in modern broiler breeding, resulting a greater genetic selection intensity for body size in CC than that in RR [60, 61]. At D42, the BW and PMW of CC was approximately 21% and 33% higher than that of RR. Typically, the broiler chickens weigh at 4–5 times that of layer chickens at D42 [62]. These findings suggested that the muscle development of broilers of CC and RR was similar. As the fundamental structural unit of muscles, it is well-known that multinucleate muscle fiber formation occurs during the embryonic period [63, 64]. The number of muscle fibers cannot be increased after hatching, and muscle development primarily depends on the hypertrophy of existing muscle fibers [65]. The CSA and MFD of muscle fibers are crucial phenotypes for assessing the developmental status of skeletal muscles [66, 67]. Therefore, we further assessed the histological characteristics of myofibers in the pectoral muscle in the two broiler breeds after DOH. The results indicated that the CSA and MFD of muscle fibers in the CC and RR groups were not significantly different. Given that CSA and MFD were measured based on the local muscle fiber field, we speculate that the pectoral muscle of the CC group possesses a higher total number of muscle fibers. When scrutinizing the genetic variations across different breeds and time periods, notable differences were observed, with the disparities between D21 and D42 being more pronounced.

Moreover, the transcriptional analysis of the two groups over time revealed a higher number of upregulated genes at D42 compared to D21. Remarkably, the PCA plot derived from RNA-seq and ATAC-seq revealed that PC2 effectively distinguishes samples based on different ages. These findings implied that the developmental regulation pattern of muscle at D42 may be more complex than at D21.

Additionally, the D21BGs of RR were enriched in terms such as myosin complex and cytoskeletal motor activity that included MYH1D, MYO1A, and ACTC1. The D42BGs in RR exhibited enrichment in defense response and immune system processes, as well as pathways such as cytokine-cytokine receptor interaction and cell adhesion molecules.

Enrichment in pathways related to apical plasma membrane, actin-based cell projection, and myosin complex was significantly observed in D21BGs of CC and RR. The key genes associated with muscle development, including *ACTC1* [68–70] and *MYH1D* [71] were identified. Studies have highlighted the pivotal role of the *MYH1D*, belonging to the *MYH* gene family, in improving the body weight and muscle production in broiler chickens [71]. In contrast, D42BGs of RR demonstrated enrichment in defense response and immune system processes, along with pathways such as cytokine-cytokine receptor interaction and cell adhesion molecules. These results suggest a more prominent muscle development process in broiler chickens at D21, coupled with heightened immune responses at D42.

The integration of RNA-seq and ATAC-seq data holds the potential to enhance the characterization of chromatin accessibility and gene expression patterns critical for the progression of pectoral muscle development in broiler chickens. Here, we did observe a certain correlation between the expression of DEGs and the accessibility of DARs, which is consistent with the results from other studies [72, 73]. By combining the results of DEGs with DARs, we identified the upregulation of *TMEM164* in the CC group at D21. *TMEM164*, a member of the transmembrane protein (*TMEM*) family [74], has been reported in the regulation of positive regulation of intramuscular fat deposition in pectoral muscle of chicken [75]. But there were no previous studies in *TMEM164* and muscle development. Previous research has shown that *TMEM182*, also belonging to the *TMEM* family, acts as an inhibitor in skeletal muscle development, growth, and regeneration [76]. However, further studies are necessary for a comprehensive understanding of the specific muscle development regulatory effects of *TMEM164*.

The gene expression level of *ACTC1*, a cardiac muscle protein that pivotal in cellular division, migration, and vesicular transport [77], was significantly higher at D21 than at D42 in broiler chickens. *ACTC1* has been identified in both bovine and porcine skeletal muscle tissue and muscle cells [78, 79], signifying its enrichment in the pathway related to muscle contraction. In addition, studies have shown that *ACTC1* plays a crucial supportive role in the maintenance of human Pax7^+^ myogenic progenitor cells and the promotion of muscle regeneration after injury [80]. The results found in our study also provide additional support for the crucial role of *ACTC1* in skeletal muscle development. Neuregulin 1 (*NRG1*), a trophic factor produced by nerves and muscles [81], belongs to the epidermal growth factor (*EGF*) family and facilitates communication between motor axons and muscles, as well as among different muscle fibers [82]. The findings of this study indicate that the expression of *NRG1* in broiler chickens was approximately three times higher at D21 compared to D42, suggesting increased neuromuscular activity in broiler chickens at D21. Furthermore, substantial research has highlighted the connection between disturbances in cholesterol synthesis and impairments in skeletal development, emphasizing the crucial role played by cholesterol synthesis in the development of skeletal muscles [83, 84]. *FDPS* and *CETP*, related to cholesterol synthesis, were also identified at D21 in this study. *FDPS* plays a pivotal role in the cholesterol synthesis of the body, primarily by catalyzing the conversion of isopentenyl diphosphate into farnesyl pyrophosphate [85].

The transforming growth factor-β (*TGF-β*) family constituents exert significant influences on skeletal muscle cell proliferation and differentiation across various animal, with *TGFB3* being particularly essential for proper muscle development [86, 87]. It is noteworthy that broiler chickens exhibit sustained accelerated muscle growth at D42, which may be closely related to the elevated expression of *TGFB3*. Importantly, the study revealed the presence of several genes associated with muscle regeneration, such as *FOS* and *MUSTN1*, in broilers at D42. Research has shown that *FOS* plays a crucial role in initiating key stem cell functions including cell cycle entry, proliferative expansion, and muscle regeneration [88]. The function of *MUSTN1* in skeletal muscle physiology is multifaceted, with the knockdown of *MUSTN1* has been shown to induce changes in myofiber types [89] and to exert influences on both myoblast differentiation and muscle regeneration processes [90]. Myoblast differentiation is impaired and the fusion of myoblasts fails to occur with the absence of *MUSTN1*. In-depth vivo experiments have demonstrated that *MUSTN1* functions as an early marker of myoblast fusion, playing a role in skeletal muscle development and regeneration [90]. Furthermore, inhibition of *MUSTN1* expression impairs myoblast differentiation by targeting *MYOD* and *MYOG* [91]. Interestingly, our H&E staining results of muscle results showed that numerous myonuclei within the muscle fibers of broilers during the growth period were located centrally rather than at the periphery, suggesting a state of continuous muscle repair [92, 93]. This further indicates that intense muscle damage may coincide with rapid muscle growth until D42. To ensure continuous muscle mass growth, the organism responds by upregulating *FOS* and *MUSTN1*, facilitating repair and achieving a stable state of muscle differentiation in the body.

## Conclusion

This research provides a high-resolution exploration of the transcriptome and chromatin accessibility landscapes of pectoral muscle in broiler chickens. Our study explores the developmental dynamics of the pectoral muscle, focusing on various dimensions and the results underscore that temporal differences outweigh those observed in the breed dimension. Moreover, our findings illuminate that the rapid growth observed in broiler chickens induces continuous muscle damage followed by subsequent regeneration. Key candidate genes, including *ACTC1*, *FDPS*, *NRG1*, *TGFB3*, *MUSTN1* and *FOS*, were identified to play crucial roles in the rapid post-hatching development of broiler chickens. Overall, our research contributes to a deeper understanding of the regulatory mechanisms governing late-stage muscle development in broiler chickens. Additionally, the identification of key candidate genes lays a foundation for potential future studies delving into the functional aspects of muscle development.

### Supplementary Information

**Additional file 1: Table S1.** Experimental sample size of each analysis. **Table S2.** Summary of sequencing quality and reads alignment statistics of RNA-seq data. **Table S3.** Summary of sequencing quality and reads alignment statistics of ATAC-seq data. **Table S4.** Growth performance of CC and RR from DOH to D42. **Table S5.** Differentially expressed genes profiles between D21 and D42 of CC. **Table S6.** Differentially expressed genes profiles between D21 and D42 of RR. **Table S7.** Differentially expressed genes profiles between CC and RR at D21. **Table S8.** Differentially expressed genes profiles between CC and RR at D42. **Table S9.** Differential accessible regions profiles between D21 and D42 of CC. **Table S10.** Differential accessible regions profiles between D21 and D42 of RR. **Table S11.** Differential accessible regions profiles between CC and RR at D21. **Table S12.** Differential accessible regions profiles between CC and RR at D42. **Table S13.** The DEGs carrying with DARs.

**Additional file 2:** **Fig. S1.** KEGG pathway enrichment analysis on the differential gene. **a** Function analysis on differential genes between CC and RR at D21 and D42. **b** Function analysis on differential genes between D21 and D42 of CC and RR. **Fig. S2.** Chromatin accessibility analysis of CC and RR at D21 and D42. **a–c** Fragment insert size of CC-D42, RR-D21 and RR-D42 groups. **d** Number of peaks in the four groups. **e** Heatmaps of differential accessible regions (DARs). **f** The relationship between differentially expressed genes (DEGs) and DARs.

## Data Availability

The datasets generated and analyzed during the current study are not publicly available. They can be made available from the corresponding author upon reasonable request.

## Abbreviations

ACTC1: Actin alpha cardiac muscle 1
ATAC-seq: Assay for transposase accessible chromatin with high-throughput sequencing
BW: Body weight
CBGs: CC-biased DEGs
CC: Cornish
CETP: Cholesteryl ester transfer protein
CSA: Cross-sectional area
DOH: Day of hatching
D7: Day 7 after hatch
DARs: Differential accessible regions
DEGs: Differentially expressed genes
D21BGs: D21-biased DEGs
D42BGs: D42-biased DEGs
FDPS: Farnesyl diphosphate synthase
FOS: Fos proto-oncogene, AP-1 transcription factor subunit
GO: Gene ontology
GWAS: Genome-wide association studies
HBA1: Hemoglobin subunit alpha 1
HBBA: Hemoglobin subunit epsilon 1
H&E: Hematoxylin–eosin
KEGG: Kyoto encyclopedia of genes and genomes
MFD: Muscle fiber density
MUSTN1: Musculoskeletal, embryonic nuclear protein 1
NRG1: Neuregulin 1
PCA: Principal component analysis
PMW: Pectoral muscle weight
RBGs: RR-biased DEGs
RNA-seq: High-throughput paired-end RNA sequencing
RR: White Plymouth Rock
TGFB3: Transforming growth factor beta 3
TMEM164: Transmembrane protein 164
TSS: Transcriptional start site
WNT9A: Wnt family member 9A
