# Supplementary figures and images for: Transcriptomic and epigenomic landscapes of muscle growth during the postnatal period of broilers
Source: J Anim Sci Biotechnol. 2024 Jul 4;15:91. doi: 10.1186/s40104-024-01049-w (PMC11223452; doi:10.1186/s40104-024-01049-w)

**a**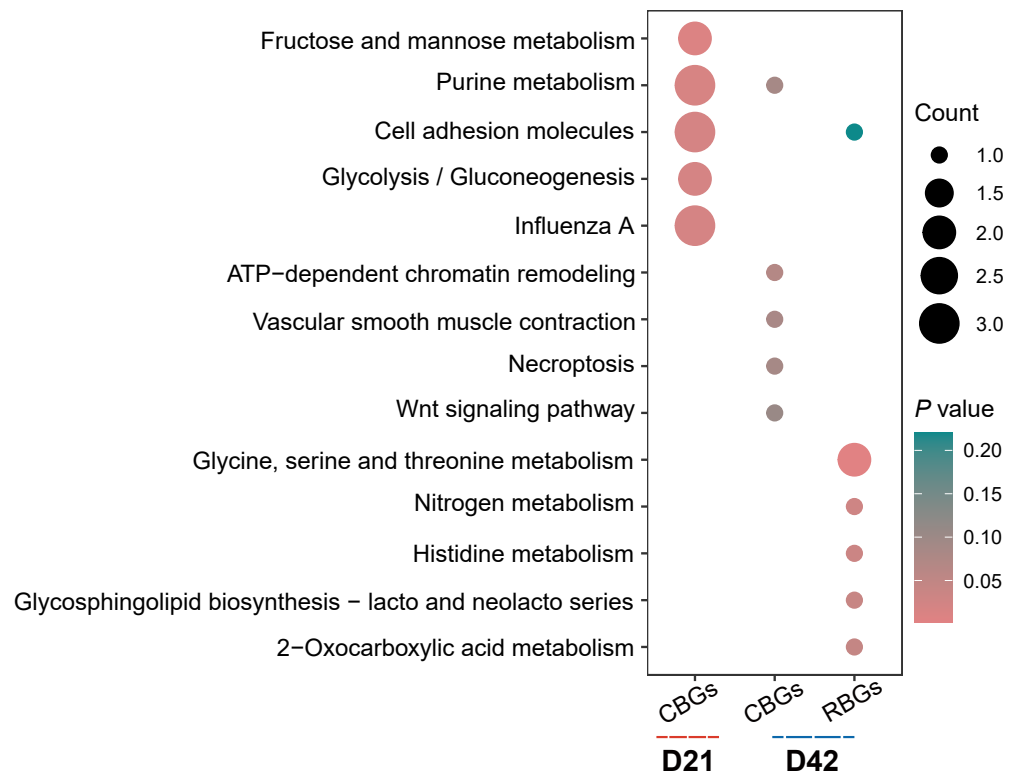**b**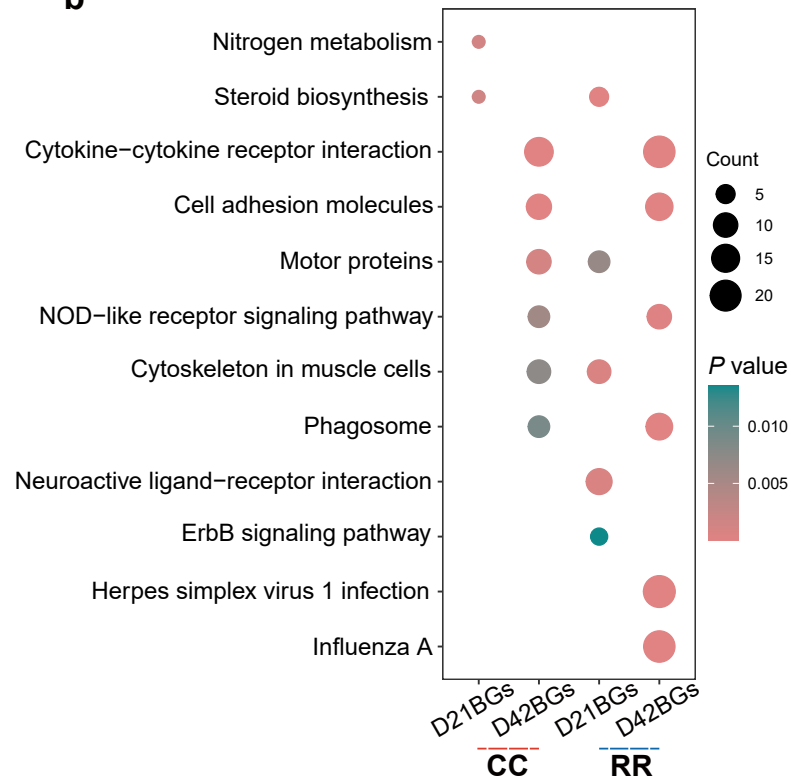

Supplement: Supplementary file 2 — Additional file 2: Fig. S1. KEGG pathway enrichment analysis on the differential gene. a Function analysis on differential genes between CC and RR at D21 and D42. b Function analysis on differential genes between D21 and D42 of CC and RR. Fig. S2. Chromatin accessibility analysis of CC and RR at D21 and D42. a–c Fragment insert size of CC-D42, RR-D21 and RR-D42 groups. d Number of peaks in the four groups. e Heatmaps of differential accessible regions (DARs). f The relationship between differentially expressed genes (DEGs) and DARs. [file 40104_2024_1049_MOESM2_ESM.zip › Figure S1.pdf]

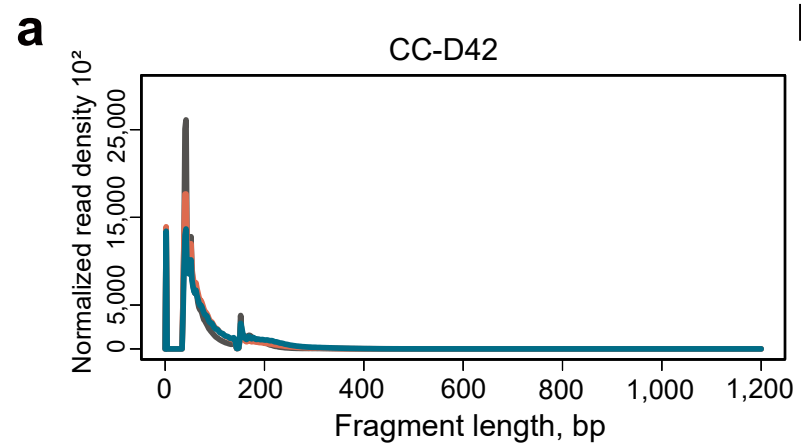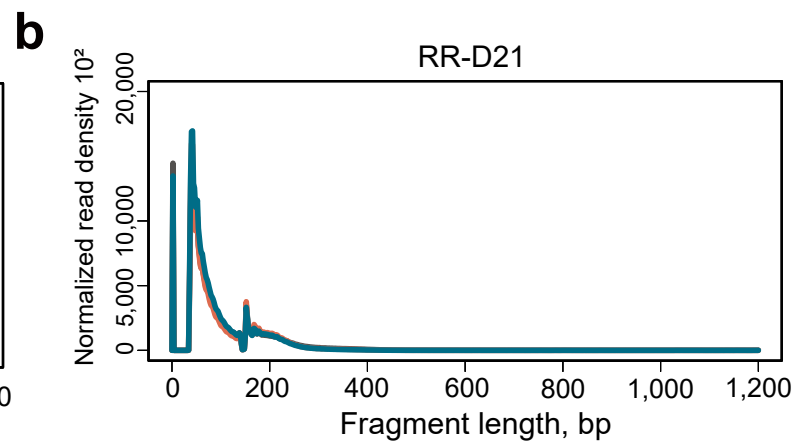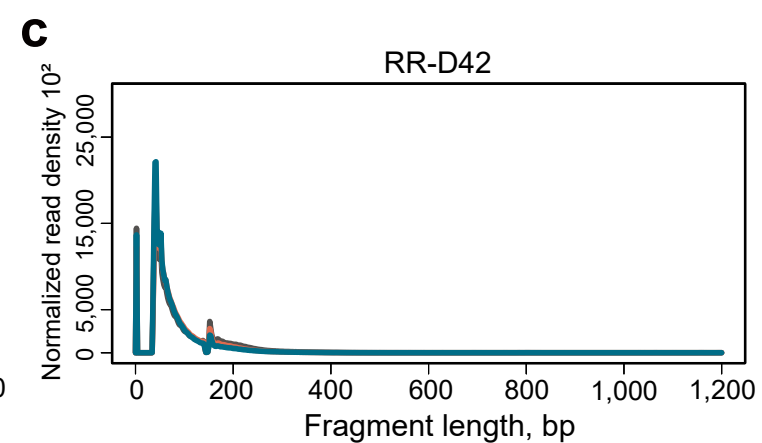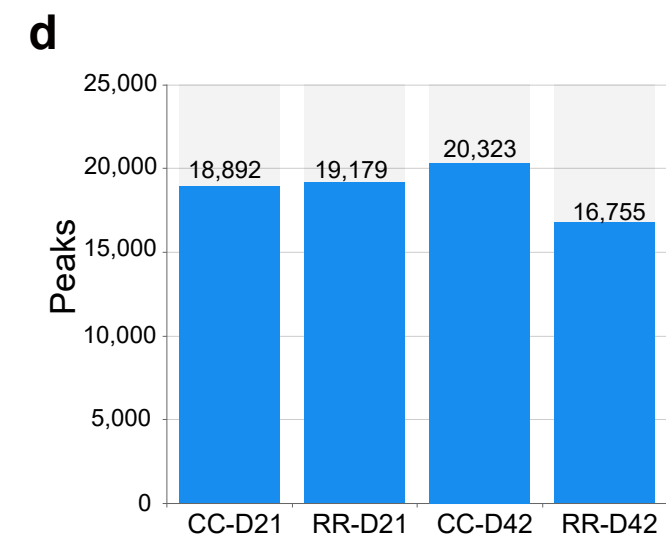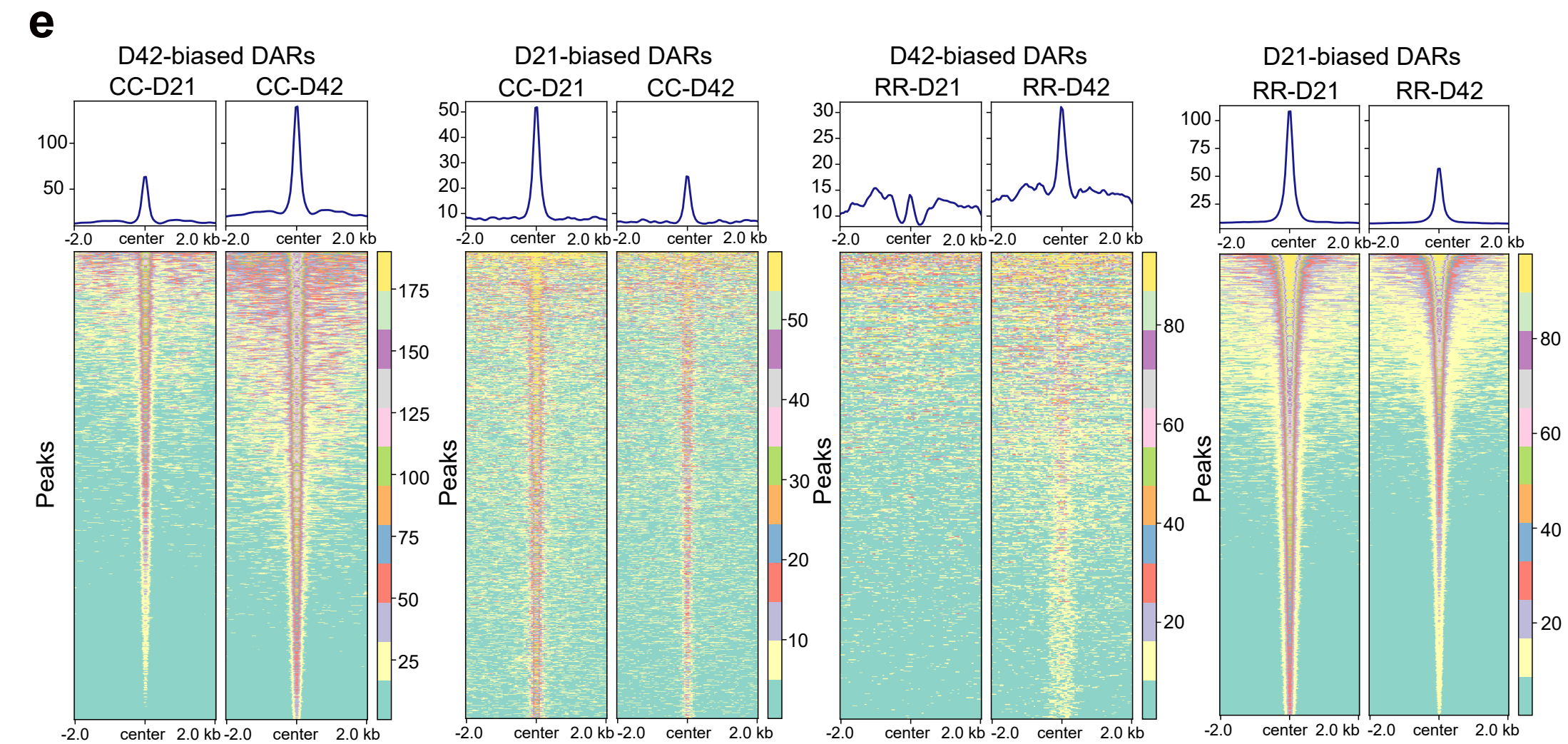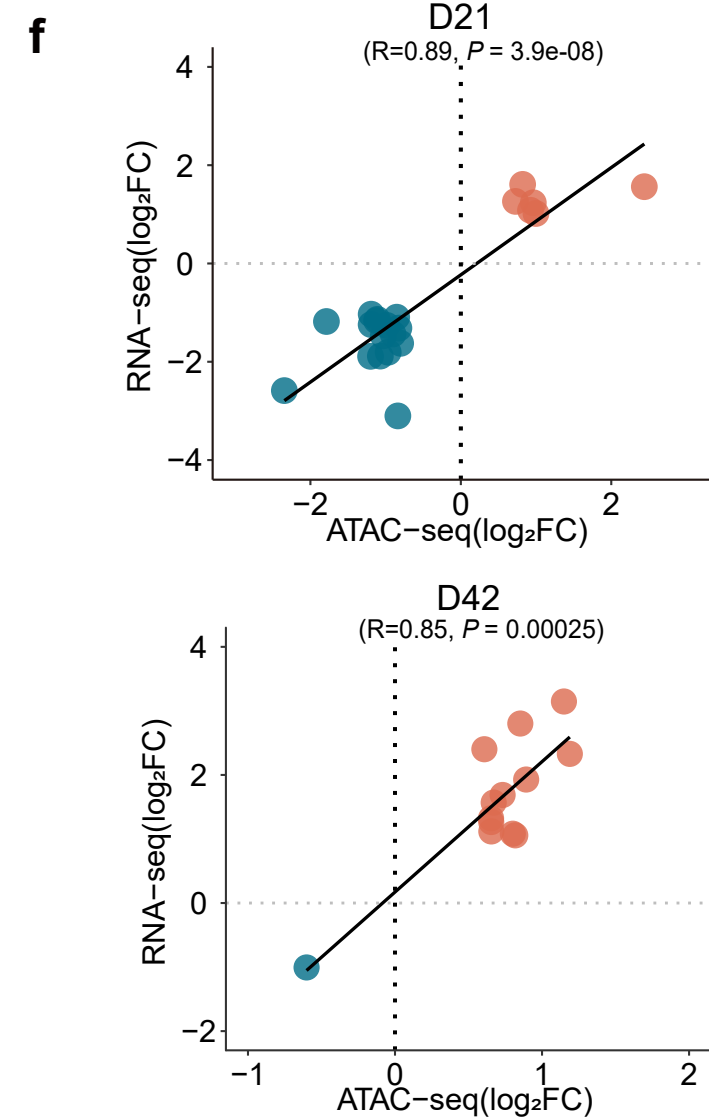

Supplement: Supplementary file 2 — Additional file 2: Fig. S1. KEGG pathway enrichment analysis on the differential gene. a Function analysis on differential genes between CC and RR at D21 and D42. b Function analysis on differential genes between D21 and D42 of CC and RR. Fig. S2. Chromatin accessibility analysis of CC and RR at D21 and D42. a–c Fragment insert size of CC-D42, RR-D21 and RR-D42 groups. d Number of peaks in the four groups. e Heatmaps of differential accessible regions (DARs). f The relationship between differentially expressed genes (DEGs) and DARs. [file 40104_2024_1049_MOESM2_ESM.zip › Figure S2_ESM.pdf]
